# Genome Sequences of Equid Herpesviruses 2 and 5

**DOI:** 10.1128/genomeA.00119-15

**Published:** 2015-03-12

**Authors:** Gavin S. Wilkie, Karen Kerr, James P. Stewart, Michael J. Studdert, Andrew J. Davison

**Affiliations:** a MRC–University of Glasgow Centre for Virus Research, Glasgow, United Kingdom; b Department of Infection Biology, Institute of Infection and Global Health, University of Liverpool, Liverpool, United Kingdom; c Centre for Equine Virology, School of Veterinary Science, The University of Melbourne, Melbourne, Australia

## Abstract

We resequenced the genome of equid herpesvirus 2 (EHV2) strain 86/67 and sequenced the genomes of EHV2 strain G9/92 and equid herpesvirus 5 (EHV5) strain 2-141/67. The most prominent genetic differences are the dissimilar locations of the interleukin-10 (IL-10)-like genes and the presence of an OX-2-like gene in EHV5 only.

## GENOME ANNOUNCEMENT

Equid herpesviruses 2 and 5 (EHV2 and EHV5) belong to genus *Percavirus*, subfamily *Gammaherpesvirinae*, family *Herpesviridae* (1, 2). Evidence for the causation of the diseases associated with these viruses remains elusive (3), except in the case of EHV5-induced pulmonary fibrosis (4). The genome sequence of EHV2 86/67 (5, 6) has been published (7). We resequenced this strain and also sequenced EHV2 G9/92 (8) and EHV5 2-141/67 (6).

Paired-end 250-nucleotide reads were generated from the EHV2 86/67 and EHV5 2-141/67 DNA preparations that were analyzed previously (1), and from EHV2 G9/92 DNA that was isolated at the time of the study, by using an Illumina MiSeq (version 2 chemistry). The EHV2 86/67 sequence with GenBank accession no. U20824.1 (7) was corrected by making directed assemblies using Burrows-Wheeler Aligner (BWA) (9) and Tanoti (http://www.bioinformatics.cvr.ac.uk/Tanoti/index.php), and by viewing them using Tablet (10). The draft EHV2 G9/92 and EHV5 2-141/67 sequences were assembled *de novo* by using ABySS (11), improved by using GapFiller (12) and iCORN2 (13), and assessed by making directed assemblies. Regions of low coverage or containing repeats were assessed by PCR amplification and Sanger sequencing. The EHV2 G9/92 and EHV5 2-141/67 genome termini were identified by using a PCR-based method (14).

For EHV2 86/67, EHV2 G9/92, and EHV5 2-141/67, 1,617,084, 7,239,808, and 2,411,370 reads were obtained, with the majority (84, 91, and 94%) aligned (using Tanoti) with the sequences at coverage values of 1,812, 8,776, and 3,085 reads per nucleotide, respectively. The genome sizes are 184,439, 186,110, and 182,380 bp, respectively, including an unmatched complementary nucleotide at the 3′ end of each DNA strand. The slightly larger size of the EHV5 2-141/67 genome from that estimated by restriction site analysis (179 kbp) (15) is due to the absence of a 6,176-bp region from the maps. The EHV2 86/67 and EHV2 G9/92 genomes contain direct terminal repeats (TR) of 17,553 and 18,332 bp, respectively. The TR is much smaller (10 bp) in the EHV5 2-141/67 genome, in support of previous evidence (15).

The number of functional open reading frames (ORFs) was conservatively estimated to be 78 in EHV2 (one duplicated in TR) and 79 in EHV5. Six ORFs (E1, E5A, E6A, ORF51, ORF74, and E9) exhibit <80% amino acid sequence identity between the EHV2 strains. The diversity in EHV2 has been studied by restriction site analysis (16) and in two ORFs in the list above (E1 and ORF74) by sequencing (17). Seven EHV5 ORFs (E1, E3, E6A, ORF27, ORF51, E9, and E10) exhibit <40% amino acid sequence identity to their EHV2 orthologs. One EHV2 ORF (E7, encoding an IL-10-like protein) occupies a dissimilar genome location in EHV5. One EHV2 ORF (E6C) lacks an EHV5 ortholog, and two EHV5 ORFs (E6B and E11, with E11 encoding an OX-2-like protein) lack EHV2 orthologs. The largest divergent regions in the EHV5 genome are the 15 kbp at the left terminus and the 20 kbp at the right terminus.

### Nucleotide sequence accession numbers.

The EHV2 G9/92 and EHV5 2-141/67 genome sequences have been deposited in GenBank under the accession nos. KM924294 and KM924295, respectively.
